# Benchmarking spatial discrimination thresholds of two-frame motion defined forms compared to luminance and stereoscopic defined forms

**DOI:** 10.1177/03010066261453278

**Published:** 2026-06-17

**Authors:** Henrik K. Kotaniemi, Philip M. Grove

**Affiliations:** 1 1974 The University of Queensland, Australia

**Keywords:** depth, local motion, motion, objects and features, perception, shape, spatial cognition, spatial vision, stereopsis

## Abstract

Two-frame motion defined form is a type of discontinuous motion contrast rendering objects visible through short distance displacement of a region within two otherwise identical sequentially presented random-dot patterns. The resulting illusory object is transiently perceived before fading. The purpose of this study was to benchmark visual spatial acuity for two-frame motion defined form compared to luminance and disparity defined forms. Random dot patterns of equal dot density were used for all contrast types to ensure equivalence of spatial sampling. Stimulus durations in the luminance and stereoscopic conditions were set at the subjective duration (∼270 ms) of the two-frame motion defined form measured in a control experiment. In the main experiment, participants made forced choice judgements on the aspect ratios of the target defined by each of the contrasts using the method of constant stimuli. Participants indicated whether the target square was ‘wider’ or ‘taller’ as a function of the aspect ratio of the target while controlling for trial-to-trial variations in the distance between the vertical/horizontal edges of the target and the boundaries of the surrounding dots. Group mean just-noticeable differences (JNDs) for luminance and stereoscopic conditions translated to changes in aspect ratio of 4.8% and 4%, respectively. They were significantly smaller than the two-frame motion defined condition, 5.8%. Spatial acuity for two-frame motion defined forms is only slightly inferior to other contrast types when equated for spatial sampling and duration. This study is the first benchmark of visual acuity for two-frame motion defined form against luminance and disparity defined form.

A major function of vision is to identify objects in the environment by differentiating them from their backgrounds. This is achieved through various forms of contrast which consist of luminance, colour, texture, binocular disparity and motion (Regan, 2000). Luminance contrast renders form visible via differences in light intensity between object and background (Purves et al., 2001b). Colour-defined forms are visible due to the difference in wavelengths of light emitted or reflected from neighbouring regions (Mollon & Regan, 1999; Purves et al., 2001a). Texture defined forms are perceived when the pattern of components defined by another contrast type such as luminance differ from its surroundings, bringing into vision a spatial form which would not be visible through the luminance contrast alone (Julesz, 1981).

Julesz (1960) demonstrated that objects could be rendered visible by binocular disparity alone. He developed stereo image pairs of identical random dot patterns in which a small central square region was shifted slightly to one side in one eye relative to the identical region in the other eye. When viewed side-by-side, there are no objects visible in either of the images, however when they are binocularly fused, the shifted region of dots is seen as a square floating in front of or behind the surrounding dots (Julesz, 1971). Motion-defined form relies on temporal displacement of elements in one region of the visual field relative to an adjacent region to render objects visible that are otherwise indistinguishable from their surroundings. For example, a pattern of dots moving in one direction within a ‘H’-shaped area surrounded by dots that are stationary or moving in a different direction renders the ‘H’ visible (Regan, 2000; Giaschi et al., 2024).

Interestingly, motion can be simulated through sequential presentation of images such as the stereo image pairs, known as two-frame motion (e.g., Morgan & Mather, 1994). The perceived shift of the displaced dots between the two images not only creates the appearance of a body in motion but also provides a contrast cue which makes the same illusory square present in stereoscopic viewing visible (Julesz 1971; Braddick, 1974; Lappin & Bell, 1976). Early work with two-frame motion focused on identifying distinctions between proposed short and long processes in apparent motion theorised by Braddick (1974). This so-called short-range apparent motion was studied by researchers to test the conditions under which it occurred and differentiate it from continuous motion processes (Antsis, 1980; Baker & Braddick, 1982; Braddick et al., 1980; Braddick & Cleary; 1980; Cleary & Braddick, 1990; Lappin & Bell, 1976; Morgan & Mather, 1994; Snowden & Braddick, 1990). The underpinnings of differentiated short and long processes was later questioned, claiming these distinctions were largely stimulus dependent and not indicative of any separate motion processes (Watson et al., 1986, Mather & Cavanagh, 1989). Cavanagh (1992) proposed an attention-based feature tracking mechanism accounting for motion in several stimulus types. Nishida (2011) describes the development of subsequent models accounting for motion perception in two-frame motion contexts and other third-order contexts (e.g., Lu & Sperling, 1995a, 1995b, 2001). However, the development and evaluation of these models is beyond the scope of this article. Previous work on two-frame apparent motion has utilised luminance defined stimuli to study mechanisms behind motion reversal induced by inter-stimulus intervals (Mather, 2006; Mather & Challinor, 2009) and validate predictions made by motion-energy models (Challinor & Mather, 2010). The V1-MT model has been generally used as the computational building block to understand motion perception, with lower-level processing of local one-dimensional motion signals occurring in V1 and integrated into two-dimensional velocity maps at the MT (middle temporal visual area) stage, however this does not provide any explanation of form-motion (Nishida et al., 2018). McCarthy et al. (2012) found that local form from-motion interactions play a role in perception of global form by showing distortions in global form can occur through illusory local-motion signals. Other research in form from motion has focused on dorsal and ventral stream integration in visual processing of motion using Glass patterns (Donato et al., 2020). Neural models of form-motion interaction have also suggested a modulatory relationship between stereo and motion processing pathways (Beck & Neumann, 2010). Ban et al., (2012) in an functional magnetic resonance imaging study found integration of motion and disparity cues within V3B/KO (region denoted by overlap between subdivision of visual area V3 and kinetic occipital area) of the dorsal visual cortex in depth perception. Recent motion perception research has investigated causal inference models with a Bayesian approach to explain the role of motion in grouping visual elements (Penaloza et al., 2024; Shivkumar et al., 2025; Yang et al., 2021).

An enduring question that arises when considering the various forms of visual contrast is, ‘Do some forms of contrast support superior spatial acuity than others?’ A common approach to characterise the effectiveness of each of the forms of contrast is to measure performance on spatial acuity tasks for targets defined by different contrasts. Some examples include orientation discrimination for targets defined by luminance and motion (Regan & Hamstra, 1992a), and for cyclopean defined features (Regan & Hamstra, 1995), letter identification for all five forms of contrast (Nawrot et al., 1996), spatial frequency discrimination (Grove & Regan, 2002), and aspect ratio discrimination (Abbas et al., 2013; Regan & Hamstra, 1994). Recently, visual acuity tasks have been utilised to find quantitative measures of performance in perceiving motion-defined forms in basic research contexts (Sáry et al., 1994) and in clinical populations (Giaschi et al., 2024; Hayward et al., 2011; Ninghetto et al., 2024).

Regan (2000) pointed out that despite its long history, quantitative comparisons of spatial acuity between two-frame motion defined form and other contrast types are yet to be conducted. To the best of our knowledge, no quantitative comparisons have been published since then. One possible reason for this gap in the literature is an assumption that spatial acuity for two-frame motion defined form can be inferred from performance on continuous motion defined forms. However, the transient visibility of two-frame motion defined forms (Shioiri & Cavanagh, 1992) may influence discrimination threshold performance and as such suggests the spatial acuity performance for this discontinuous motion contrast should be specifically determined. Therefore, the aim of the present study was to benchmark spatial acuity for two-frame motion defined forms compared to equivalent luminance and stereoscopic (disparity) defined forms, using aspect ratio discrimination thresholds as the dependent measure.

## Method

### Stimuli and Apparatus

#### Apparatus

The experiment was conducted on a Dell Optiplex 7000 computer running Matlab (version 9.13.0.2080170) with the Psychophysics Toolbox extension (Brainard, 1997; Pelli, 1997). Participant responses were recorded via a standard keyboard. Stimuli were presented on a 23.8-inch Dell P2422H LED monitor with resolution set to 1920 × 1080 pixels and refresh rate of 60 Hz. The monitor brightness was set to maximum during the experiment. Luminance of white indexed (RGB values [255 255 255]) regions was 127.7 cd/m^2^.

#### Random-Dot Patterns

We generated random dot patterns to equate spatial sampling across all stimuli, allowing for close comparison of two-frame motion contrast with an extensively studied contrast in luminance, and a conceptually similar one in stereoscopic contrast, comparing the simultaneous presentation of stereopairs to sequentially presented pairs.

Images of random-dot patterns with rectangular forms were generated (see Figure 1) from small cells consisting of 10 × 10 elements, with a 20% dot density. That is, of the 100 elements in each cell, 80 were coloured white and 20 were coloured black, determined randomly. Generating the images from the smaller cells allowed for a more uniform distribution to the 20% density. The resulting image size was 300 × 300 elements, subtending 12.6° from a viewing distance of 65 cm, with each element subtending 2.52 arcmin.

**Figure 1.**
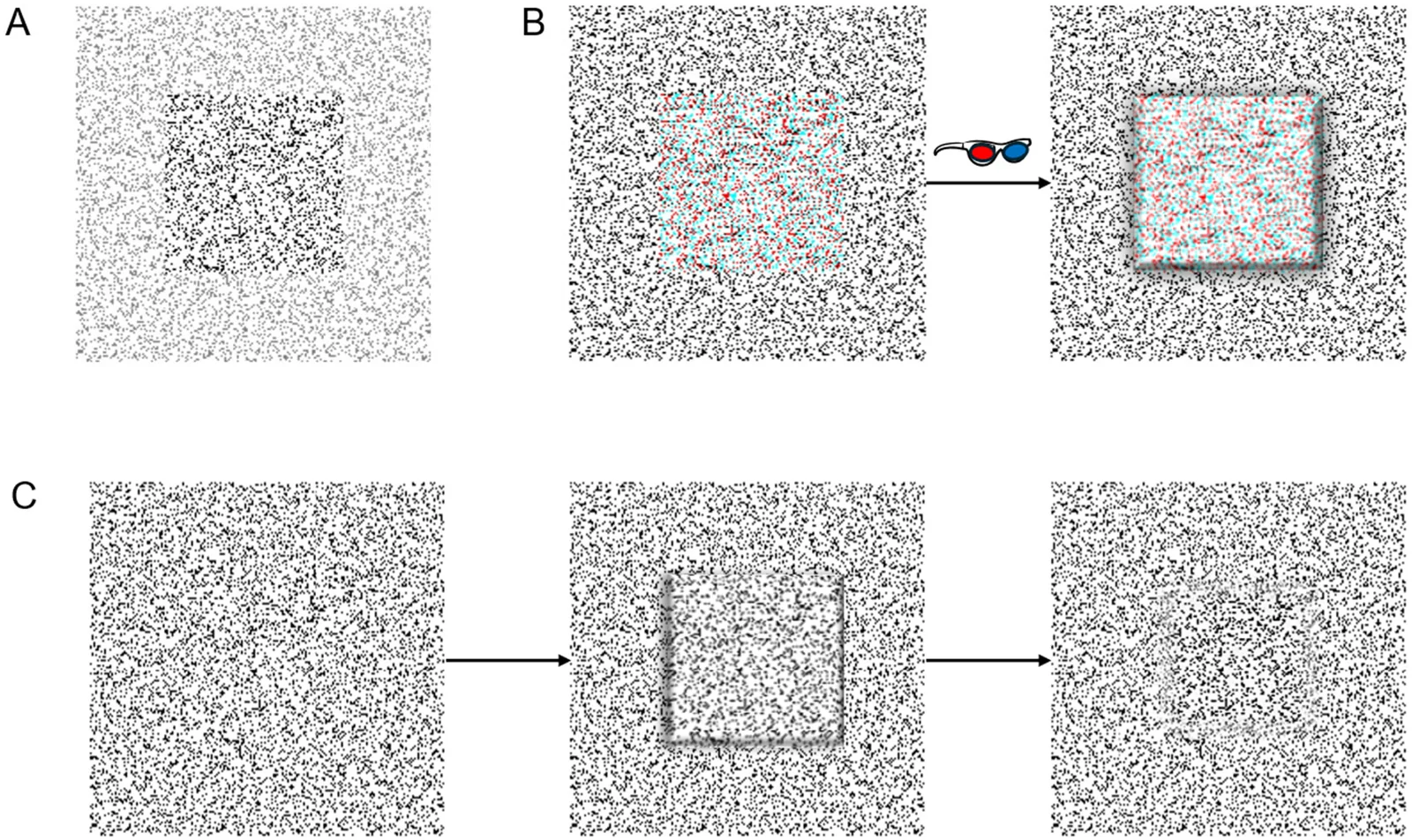
Random-dot patterns with central targets defined by three different contrast types.

(A) Luminance defined stimuli with target rectangle in the middle. (B) Stereoscopic defined form with initial image showing the stimulus appears on screen, followed by re-created effect of viewing through three-dimensional glasses. (C) Two-frame motion-defined form, stepping through initial presentation with no visible rectangle to re-created effect of sequential viewing followed by gradual disappearing immediately after.

The target rectangle area was a 100 × 100 element square region in the centre of the surrounding pattern, subtending 4.2° (for an aspect ratio of 1). The central target and background were modified to create the three different contrast defined forms, described below. Stimuli were presented against a blank white (RGB values [255, 255, 255]) background during the experiments.

#### Luminance Defined Stimuli

The luminance defined target rectangle was rendered visible through a contrast in luminance values between the dark elements in the background portion and the dark elements within the target 100 × 100 region. The light elements of the central target had RGB values of [255, 255, 255] and the dark elements had RGB values of [0, 0, 0]. The dark elements in the background were lighter in intensity (RGB values of [128, 128, 128]) to create a luminance contrast between the regions (see Figure 1).

#### Two-Frame Motion Stimuli

For the two-frame motion images, the visible form was achieved by creating a random-dot pattern and then shifting the central 100 × 100 element area laterally by two elements (5.04 arcmin), well within the upper displacement limit of 15 arcmin at which the process of two-frame motion fails found by Braddick (1974). The shifted elements of the central target replaced those in the background they moved over in this second image. The empty space left behind was filled with random elements. Two-frame motion was generated by presenting the two images in quick succession, with no delay in between. The presentation of each two-frame motion stimuli pair was counterbalanced by changing the order of the images in half of the trials, meaning in half of the trials the shift that rendered the form visible was rightward. In the other half of the trials, the shift was leftward.

#### Stereoscopic Stimuli

The stereoscopic images were created from the pairs of two-frame motion stimuli described above. The pairs of two-frame motion stimuli were combined into a single anaglyph stereogram with the target region elements of the first image being converted to red [255, 0, 0] and the shifted target region elements being converted to cyan [0, 255, 255]. With the appropriate red/cyan anaglyph glasses, the target square had crossed disparity of 5.04 arcmin relative to the surrounding dots and appeared to float in front of the background.

#### Aspect Ratios

For each contrast condition, 21 different aspect ratios were generated to create a set of images, with height to width ratios ranging evenly from 0.80 to 1.20 where a ratio of 1 was a perfect square (see Figure 2). Adjacent aspect ratios differed by 0.02, which equalled a difference of two elements between the height and width where the longest side of the target from was always 100 elements.

**Figure 2.**
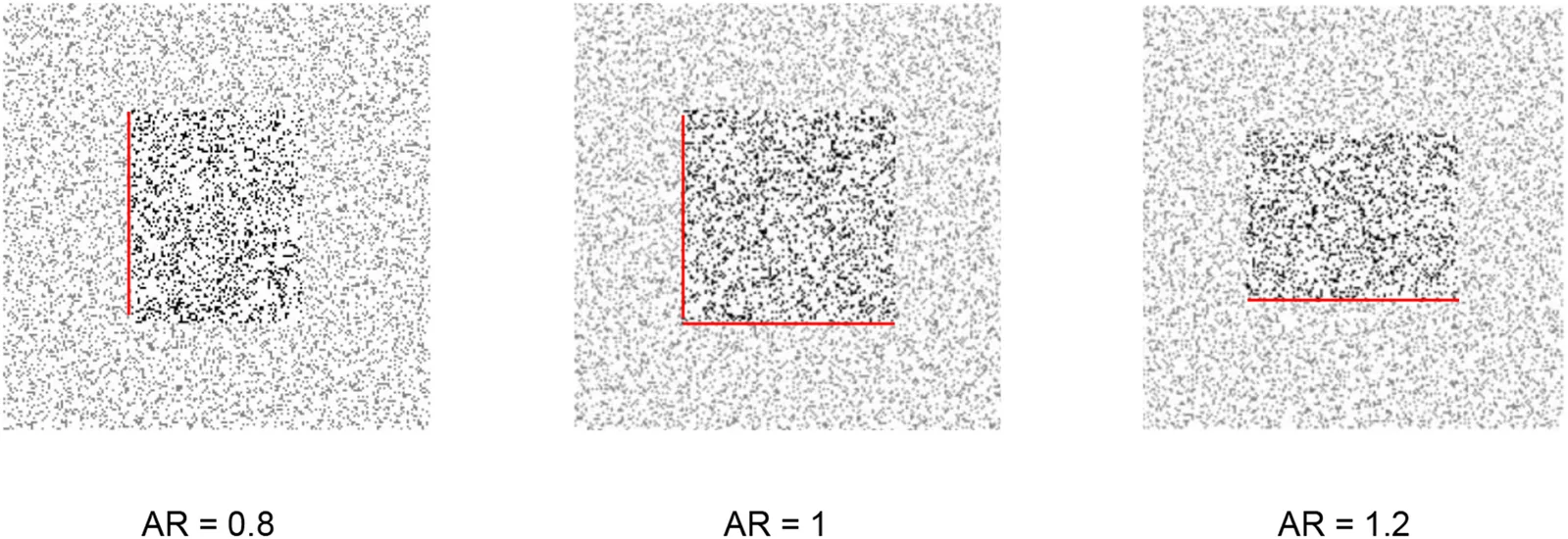
Luminance defined form stimulus examples.

*Note*. The red lines indicate the longest side of the rectangle within the random dot stereogram, with AR 0.8 being the extreme end of the ‘taller’ stimuli, AR = 1 being a perfect square and AR = 1.2 being the extreme end of the ‘wider’ stimuli.

#### Spatial Jitter of Surrounding Edges

The location of the outer vertical and horizontal edges of the background texture was randomly jittered from trial to trial by occluding the edges to varying degrees. This was done to ensure that participants were basing their responses only on the aspect ratio of the central target and not on the distance between the edges of the target and the outer edges of the surrounding dots. This was achieved by overlaying invisible white rectangles (as seen in Figure 3) over the stimulus image edges during presentation such that the inner edge of the rectangle was 160 pixels from the centre. The position of the inner edge was then randomly adjusted by vertically or horizontally shifting the occluding rectangle by one of the following values: −30, −24, −18, −12, −6, 6, 12, 18, 24 or 30 pixels.

**Figure 3.**
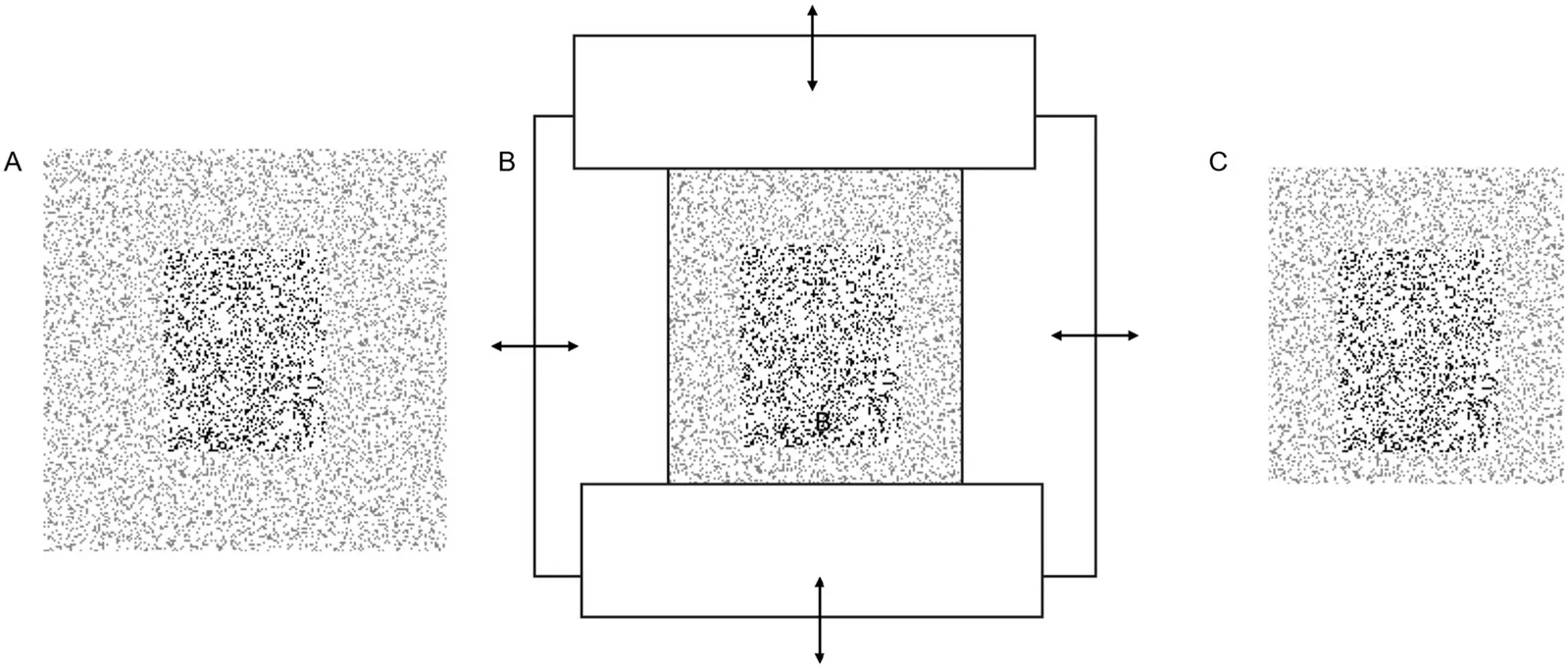
Example of spatial jitter applied to a luminance stimulus trial.

(A) Original full size 0.8 AR luminance stimulus. (B) Jitter rectangles with outlines to demonstrate borders. (C) Trial stimulus as seen by participants with jitter masking portion of random dot background.

### Preliminary Experiment—Stimulus Presentation Time

As two-frame motion defined forms are transient and can only be perceived for a short period of time, an average measure of how long the form is visible was needed to equate the duration of presentation time across stimuli. Therefore, a preliminary experiment was run to determine the perceived subjective persistence of two-frame motion defined form using a forced choice method.

#### Participants

A total of *N* = 10 participants (3 female; *Mean age* = 39.2, *SD* = 16.0) from the University of Queensland and the community were recruited. All participants reported normal or corrected to normal vision and were naïve to the purpose of the experiment. This study received ethical clearance from the University of Queensland human ethics board, approval number 2024/HE000562. Participants gave verbal consent after reading an information sheet and receiving verbal instructions from the experimenter.

#### Procedure

We used a two-interval forced choice (2IFC) method of constant stimuli procedure in which participants viewed a two-frame motion defined stimulus in one interval and a luminance defined form of varying duration in the other interval and indicated which interval contained the stimulus that was visible for longer. The 2IFC method is standard for determining an objective measure in duration comparisons in many contexts (e.g., Gao et al., 2021; Heron et al., 2012; Aaen-Stockdale et al., 2011; Hayashi et al., 2019). The presentation durations used for the luminance defined stimuli were 16.7, 116.9, 200.4, 300.6, 400.8 and 501 ms. On half of the trials the first interval contained the two-frame motion defined form, with the second image staying on the screen for 501 ms after which it was replaced by a white screen for 501 ms. The luminance defined form stimulus was then presented for one of the timing durations, after which it disappeared to a blank screen and waited for a response, after which a new trial would immediately begin. In the other half of the trials, the luminance stimulus was presented in the first interval. Each of the six duration conditions was repeated 40 times for a total of 240 randomly ordered trials.

#### Results—Preliminary Experiment

We tabulated the percentage of trials for which the luminance defined stimulus was judged as ‘longer’ for each timing. One participant was removed from the final analysis for being an extreme outlier by having a heavy skew for luminance ‘longer’ responses, resulting in the curve not passing the 50% response mark required for analysis. A cumulative Gaussian curve fit was used to provide a point of subjective equality (PSE) for each participant. The arithmetic mean PSE across the nine participants was 272.1 ms (*SD* *=* 88.3) (see Table 1). In the main experiment, we set the durations of the luminance and stereoscopic stimuli as close as possible to this value given the temporal resolution of our display (60 Hz) (Figure 4).

**Figure 4.**
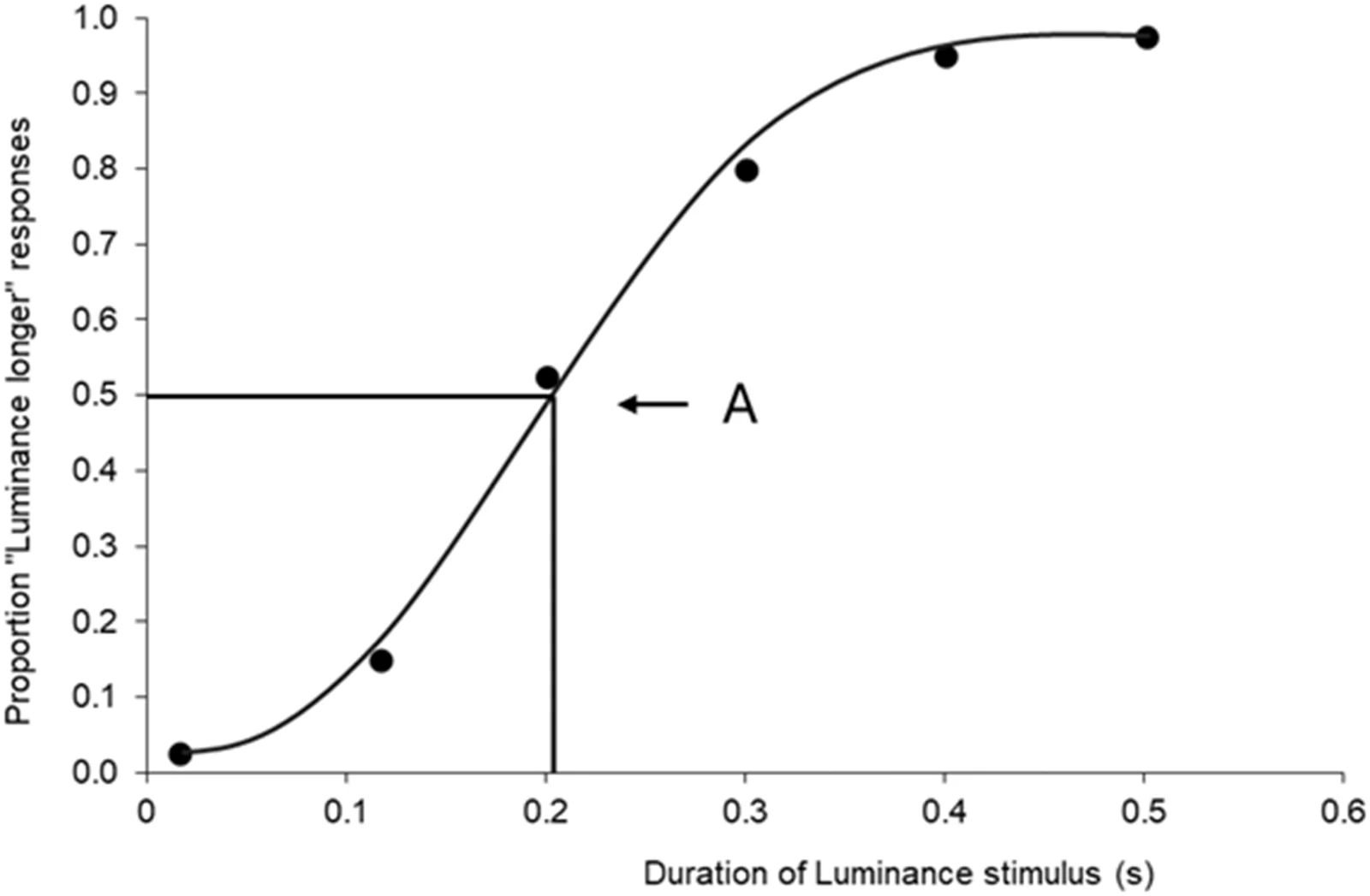
Typical participant curve fit for duration of luminance defined stimuli versus two-frame motion.

*Note*. (A) The point of subjective equality (PSE), estimating the duration perceived by the participant.

**Table 1.** PSE values for each participant included for final analysis (n = 9).

	*1*	*2*	3	4	5	6	7	8	9	*M*	*SD*
PSE (ms)	214.9	323.9	295.1	359.7	278.7	405.7	108.4	219.1	243.8	272.1	88.3

*Note.* Values expressed in milliseconds. PSE= point of subject equality.

### Main Experiment—Aspect Ratio Discrimination

#### Participants

A total of *N* = 25^1^ (14 female; *Mean age* = 33.9, *SD* = 13.6) were recruited from the community and the University of Queensland School of Psychology first year research participation scheme with course credit being rewarded for their participation. All participants reported normal or corrected to normal vision. They were all naïve to the purpose of the experiment. Participants gave verbal consent after reading an information sheet and receiving verbal instructions from the experimenter. A verification was done before formal testing with a demonstration of a sample stereogram to ensure participants had stereoscopic vision and a sample of the two-frame motion to ensure the effect was visible and the form identifiable.

#### Procedure

Participants sat in front of the screen at a viewing distance of 65 cm and were asked to make a forced choice decision on the aspect ratio of the rectangular form by responding whether it appeared to be taller or wider. The conditions were separated into three different blocks: luminance, two-frame motion and stereoscopic. Block and trial order were randomised for each participant.

For luminance and stereoscopic conditions, the stimulus remained on screen for 283 ms (17 frames at 60 Hz) after which it disappeared to a blank screen awaiting a response, after which a new trial began. For the two-frame motion conditions, the second image was presented immediately after the first which remained on screen for 300 ms, disappearing to await response before a new trial began. Each stimulus was presented 20 times with each of the three blocks consisting of 420 trials as 21 aspect ratios were used. The broad range of aspect ratios was employed to provide ample coverage for individual differences. Participants completed a total of 1260 trials in 30 to 40 min with regular breaks between blocks. If necessary, participants could rest within in a block by withholding their response until ready.

#### Data Analysis

The mean differences in the just-noticeable difference (JND) for the three contrast conditions were analysed in a repeated measures analysis of variance (ANOVA). Mauchly's Test of Sphericity showed that the assumption of sphericity had not been violated, χ^2^(2) = 1.61, *p* = .447.^2^

#### Results—Aspect Ratio Discrimination

The responses were tabulated as the number of wider responses as a function of aspect ratio from 0.80 to 1.20. A cumulative Gaussian curve was fitted to the data (see Figure 5) and the JND was determined for each participant in each of the contrast types.

**Figure 5.**
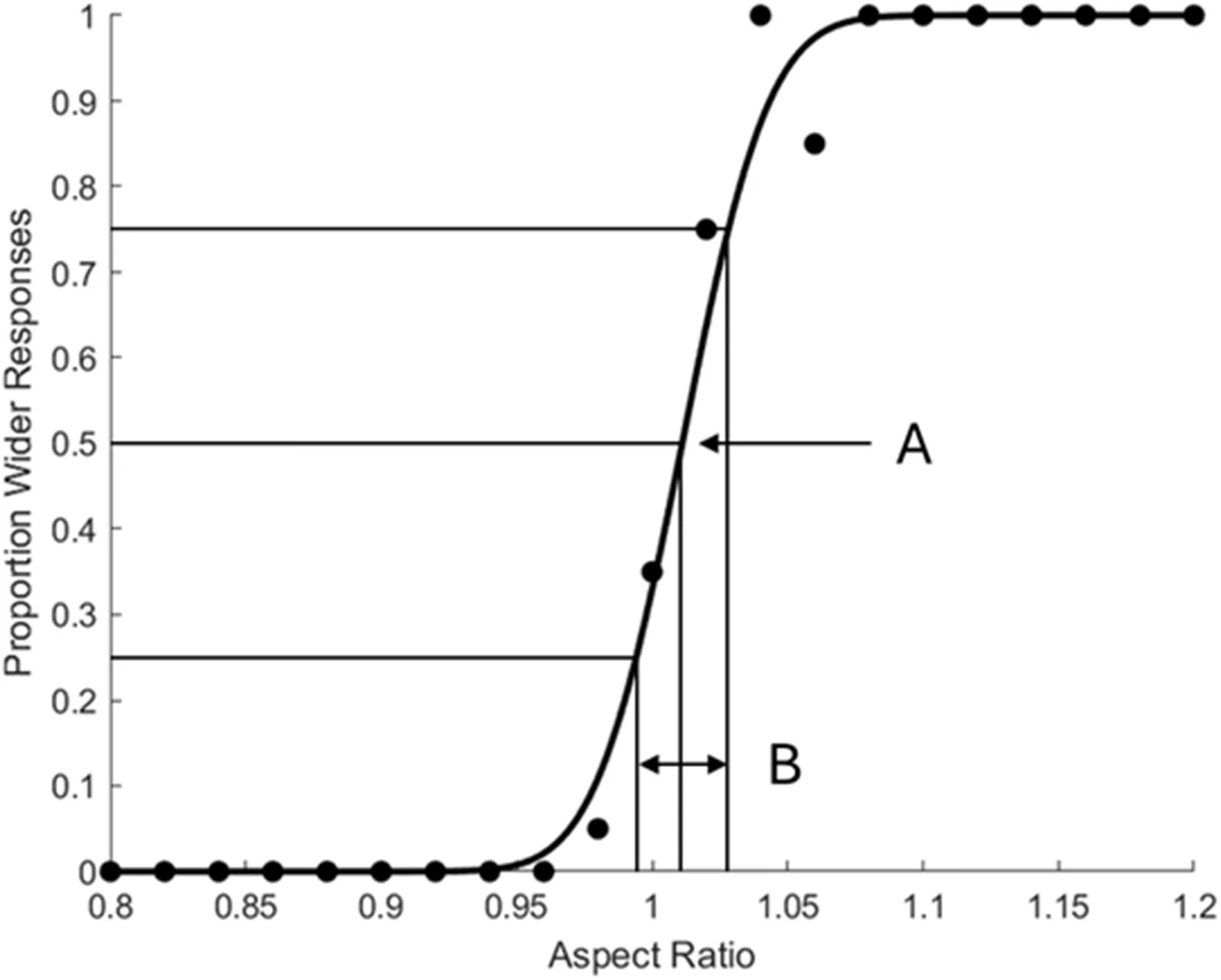
Psychometric curve for a typical participant.

*Note*. The graph demonstrates the curve fit for a typical observer from the stereoscopic condition. (A) The PSE indicated by the 50% point. (B) The aspect ratio represented by the 25% to 75% response region using the calculated curve indicated by arrows known as interval of uncertainty, where IU/2 = JND. JND= just-noticeable difference; PSE= point of subject equality.

Using participants’ JNDs as the units of analysis, a repeated measures ANOVA was conducted to assess the relationship between contrast type and JND. It was revealed that there was a significant effect of contrast type on discrimination thresholds *F* (2,48) = 11.78, *p* < .001, ηp^2^ = .329, with means and standard deviations reported in Table 2.

**Table 2.** Descriptive statistics of the discrimination thresholds (JND) for the three contrast types.

Contrast type	*M*	*SD*	Minimum	Maximum
Luminance	0.05	0.018	0.031	0.12
Two-frame motion	0.062	0.021	0.040	0.13
Stereoscopic	0.042	0.019	0.017	0.086

*Note*. All values expressed in units of aspect ratio. The mean values indicated the JND amongst the participants for each condition, indicating the average required change in aspect ratio for a difference to be reliably perceived. JND= just-noticeable difference.

Pairwise comparisons with a Bonferroni correction (α = .017), showed a significant difference between luminance and two-frame motion, *t*(24) = −3, *p* = .011, indicating that luminance provided better performance for aspect ratio discrimination than two-frame motion, while no significant difference was found between luminance and stereoscopic conditions though the stereoscopic condition yielded the lowest mean JND. A significant difference was also found between two-frame motion and stereoscopic conditions, *t* (24) = 4, *p* < .001, indicating that the stereoscopic condition yielded better performance compared to two-frame motion in aspect discrimination (see Figure 6).

**Figure 6.**
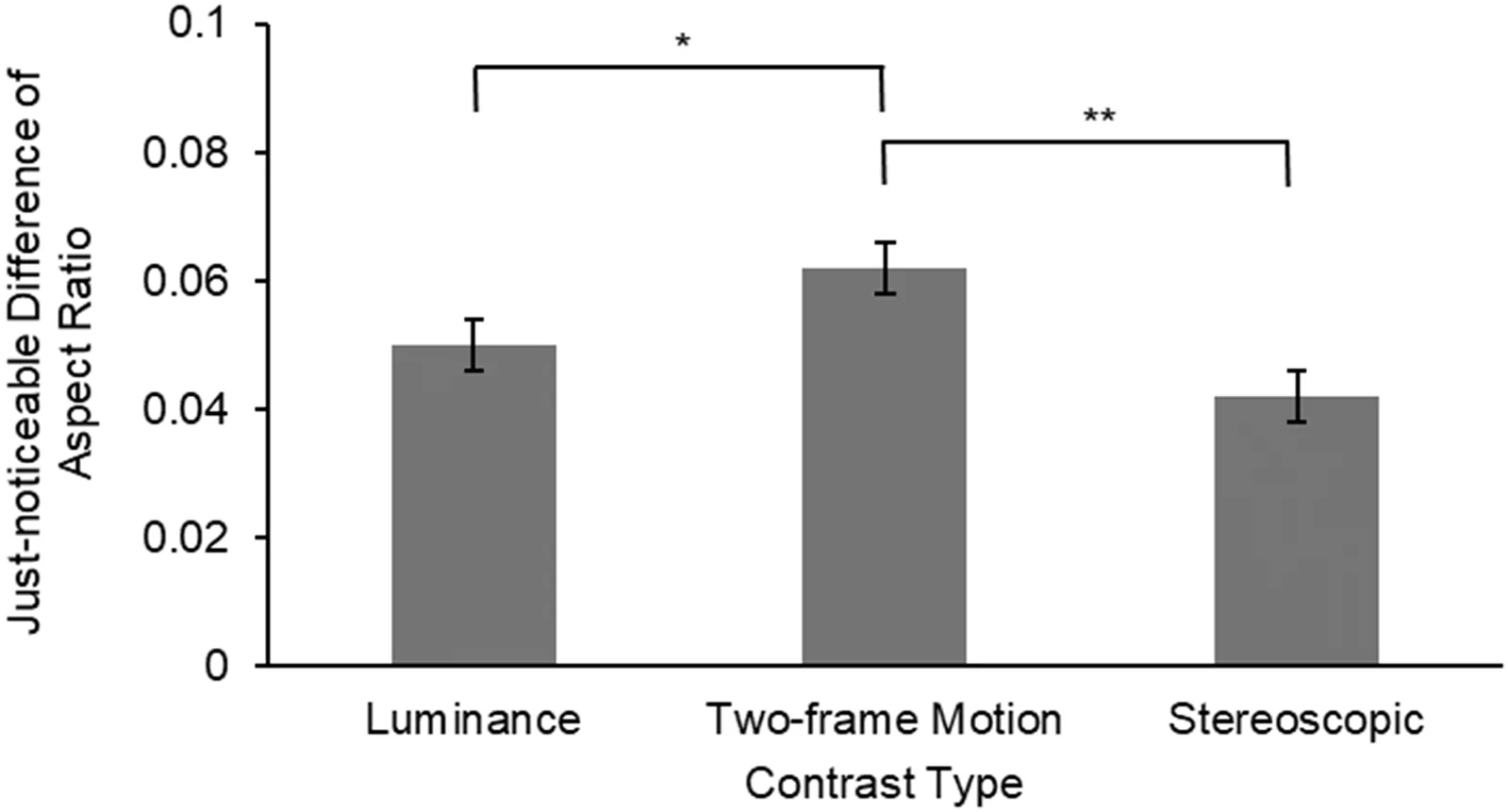
Just-noticeable difference in aspect ratio as a function of contrast type.

*Note*. Group mean just-noticeable differences for each contrast type, with error bars representing standard error. * *p* < .05; ** *p* < .001.

Group mean PSEs and associated 95% confidence intervals are shown for each condition in Table 3. The horizontal motion in the two-frame condition is a possible confound that could bias the PSE in the two-frame condition towards more ‘wider’ judgements. Inspection of the 95% confidence intervals indicates a slight bias in the two static conditions as these intervals do not contain 1. The two-frame motion confidence interval does contain 1 and therefore shows no bias.

**Table 3.** Group mean point of subjective equality for the three contrast types.

Contrast type	*M*	*SD*	95% Confidence interval	
			Lower	Upper
Luminance	1.02	0.038	1.0045	1.0361
Two-frame motion	1.01	0.035	.9967	1.0256
Stereoscopic	1.02	0.029	1.0120	1.0355

*Note.* All values expressed in units of aspect ratio. The mean values indicate the average PSE amongst the participants for each condition. PSE= point of subject equality.

To confirm the performance was due exclusively to aspect ratio judgements and not due to judgements of the relative position of the edges of the target and the edges of the surround, the aspect ratio responses for each participant were tabulated as a function of the respective jitter values in both the −x and −y dimensions for each condition and fitted with a linear regression function (see Figure 7 for an example).All slopes resembled flat lines with little to no gradient. Had the participants used edge position as a cue in determining aspect ratio we would have expected to observe steep functions, but the flat lines indicated there was no relationship between the edges of the background image in the participants judgements.

**Figure 7.**
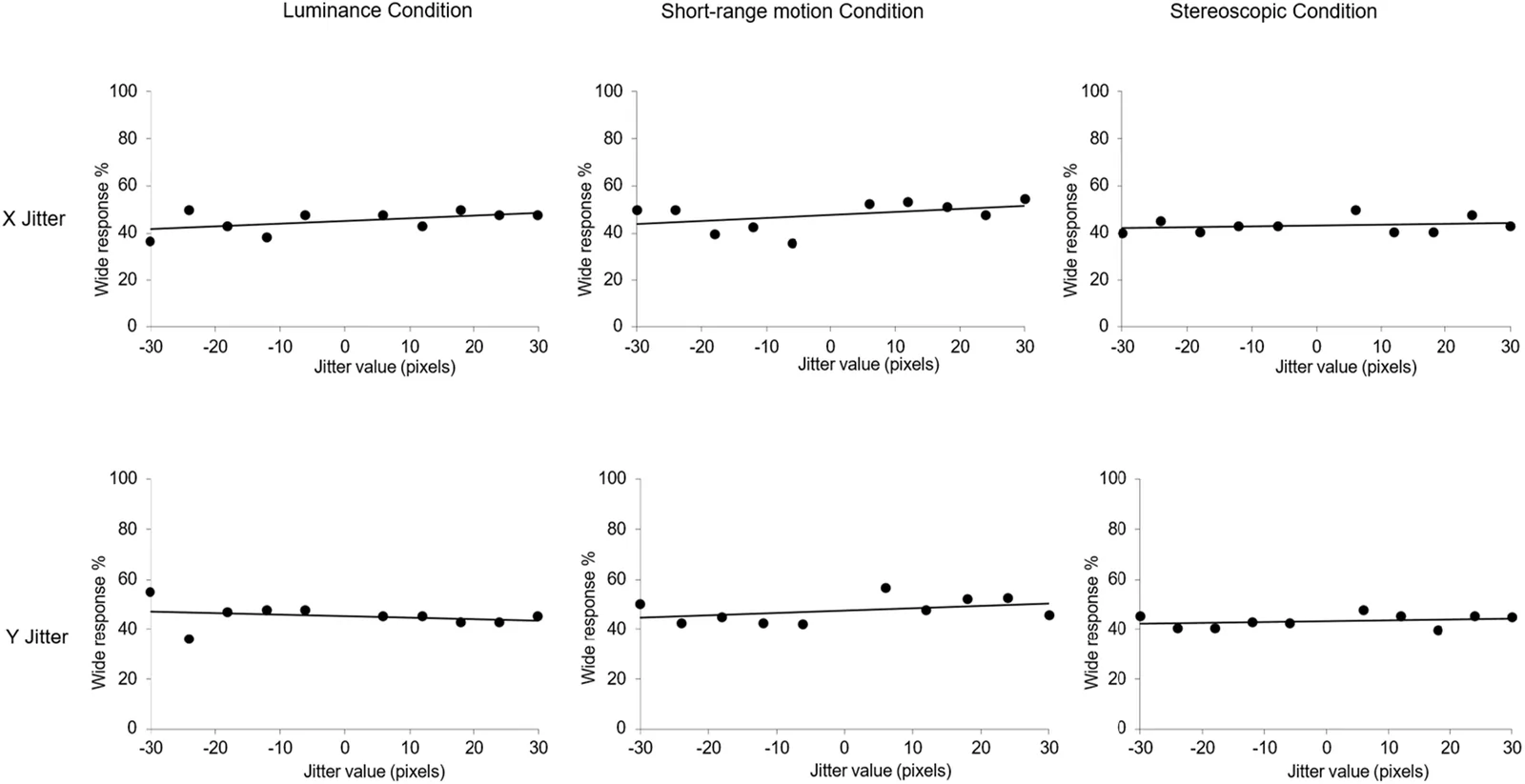
Jitter value gradients from a typical participant.

*Note*. The gradients for both x and y jitter values are plotted for their respective conditions for a typical participant.

We compared the slope of the regression functions to the respective condition's aspect ratio psychometric curve. In each condition, all 25 participants had a steeper gradient for the aspect ratio than for the jitter data. Testing the null hypothesis that the gradients obtained from aspect ratio responses or the jitter gradients would be steeper with equal probability (*p* = .05). The probability of 25 out of 25 steeper slopes in the aspect ratio responses differs significantly from chance (binomial test, *p* < .001), indicating that participants responded to trial-to-trial variations in aspect ratio and not to variations in separation with the border of surrounding dots.

## Discussion

The aim of the present study was to benchmark the levels of spatial acuity in humans for two-frame motion defined forms in comparison with luminance and stereoscopic defined forms that were equated for spatial sampling and duration.

### Preliminary Experiment Duration Value

To equate for perceived duration, a preliminary experiment was conducted to estimate how long the two-frame motion defined form remains visible. The average PSE value of 272.1 ms was higher than previously reported by Shioiri and Cavanagh (1992) (130 ms) and present as a potential limitation of the article. The methodology employed by Shioiri and Cavanagh (1992) differed from ours in at least three ways. First, Shioiri and Cavanagh tested three experienced psychophysical observers, whereas we tested ten naïve observers. Second, participants in their experiment completed a missing element detection task in which participants had to identify which of several targets rendered visible in the first interval did not appear in the second interval. The display size was 15°. In our experiment, participants attended to a single target in central vision. Third, motion in the background was in the opposite direction to that of the targets whereas the dots were static in the background of our stimuli. The differences in duration of visibility of the two-frame motion defined form in these two studies could be attributed to any or all of these discrepancies. Nevertheless, our aim was not to test Shioiri and Cavanagh's results but to equate all three stimulus types for perceived duration.

### Main Experimental Findings

Performance in discriminating two-frame motion defined aspect ratios was found to be poorer compared to the luminance and stereoscopic conditions. To illustrate the relative difference in aspect ratio represented by the JND values, a mean JND of 0.062 for two-frame motion defined forms indicates it would take a minimum change of 5.8% in aspect ratio before a difference from squareness could reliably be perceived. For luminance defined forms, the mean JND of 0.05 translates to a change of 4.8%, similar to that for stereoscopic defined forms, mean JND of 0.042 or a change of 4%. Though the luminance and stereoscopic contrasts were found to have lower JNDs than the two-frame contrast, the difference only translated to a 1% to 2% advantage. This demonstrates that two-frame motion contrast is still comparable to luminance and disparity contrast when matched for spatial sampling and duration.

Though the transient two-frame motion defined form did not provide the same precision for discrimination as a persistent static stereoscopic defined form, the difference in JND being less than 2% suggests that two-frame motion defined form is comparable to persistent stereoscopic defined form when equated for spatial sampling and duration. This similar performance from identical stimuli which only differ in mode of presentation that produce the same visual phenomena suggests similar correlation processes for both forms of contrasts. This would speak to the neural relationships found between stereo and motion processing (Ban et al., 2012; Beck & Neumann, 2010). Future experiments are planned to probe the relationship between the two phenomena investigating if incremental decorrelating the images for both contrast conditions cause the effect to breakdown in a comparable fashion.

Our findings that aspect ratio discrimination thresholds were lower for luminance defined targets than for two-frame motion defined targets are consistent with Regan and Hamstra (1992a) who found stimulus durations of less than 1s resulted in larger differences in discrimination thresholds between luminous contrast and motion contrast, though they were not equated for duration. The results from the current study show even when luminance defined form was equated for duration with the two-frame motion defined form in our study, discrimination thresholds were still lower for the former.

We did not find a significant difference in thresholds between luminance defined form and stereoscopically defined form. This differed slightly from the findings of Grove and Regan (2002) where spatial frequency discrimination thresholds were lower for luminance defined gratings than for disparity defined gratings. However, the tasks were different in the two studies and the participants in Grove and Regan's study were trained in psychophysical methods, and this could be reflective of the effect experience level can have when measuring for small differences in performance on psychophysical tasks.

Although we found no bias in the aspect ratio judgements in the two-frame motion condition, one limitation in this study is that the luminance and stereoscopic conditions were entirely static. In future experiments the target squares in the luminance and stereoscopic conditions should shift horizontally in the same manner as in the two-frame motion condition. Further experiments might investigate how cycling the two-frame sequence and extending the duration of visibility of the two-frame motion stimulus impacts spatial acuity.

## Conclusion

This study has provided the first quantitative comparison of spatial acuity across two-frame motion contrast, luminance and stereoscopic contrasts. Initial testing established estimated duration of visibility for two-frame motion defined forms to match presentation times of similar stimulus formats in an aspect ratio discrimination task. It was found that when equated for spatial sampling and duration, the luminance contrast and stereoscopic contrast yielded lower discrimination thresholds than two-frame motion contrast. Under these conditions there was also no significant difference between the performance in luminance contrast and stereoscopic contrast conditions. The use of the same two-frame stimulus, sequentially presented to generate two-frame motion contrast and simultaneously presented to generate stereoscopic contrast also provided comparison between sequential and simultaneous viewing of the same stimuli suggesting a similar correlational computation involved in their respective contrasts, which could be explored in future experiments.

## Supplemental Material

sj-docx-1-pec-10.1177_03010066261453278 - Supplemental material for Benchmarking spatial discrimination thresholds of two-frame motion defined forms compared to luminance and stereoscopic defined forms Supplemental material, sj-docx-1-pec-10.1177_03010066261453278 for Benchmarking spatial discrimination thresholds of two-frame motion defined forms compared to luminance and stereoscopic defined forms by Henrik K. Kotaniemi and Philip M. Grove in Perception

## Notes

1. The original rationale for the sample size was simply to be substantially larger than the samples of previous studies in this area. The previous literature for similar psychophysics experiments as cited in the introduction (e.g., Grove & Regan, 2000; Regan & Hamstra, 1992a; Regan & Hamstra, 1994) employed samples of two or three expert psychophysical observers. We reasoned that a sample of 25 naïve participants was sufficient. Nevertheless, a post hoc power analysis using G*Power (Faul et al., 2007) with an alpha level set at .05, power set at .95 and our observed effect size (eta-squared =.329) indicated a required sample of 18.

2. The units of analysis for the repeated measures ANOVA were the JNDs derived from the Gaussian fits of individual psychometric functions. Therefore, they are model-derived parameter estimates rather than raw data and so a standard test for normality is not appropriate in this case and was not conducted.
