# Supplementary material for: Benchmarking spatial discrimination thresholds of two-frame motion defined forms compared to luminance and stereoscopic defined forms
Source: Perception. 2026 Jun 17;55(11):1018–32. doi: 10.1177/03010066261453278 (PMC13691234; doi:10.1177/03010066261453278)
Supplement: Supplementary file 1 [file sj-docx-1-pec-10.1177_03010066261453278.docx]

# Appendix

Curve fit script outputs for Preliminary experiment

Raw scores for Preliminary experiment

JND Results for Main Experiment

| Jitter | Lum_X | Lum_Y | TWO-FRAME_X | TWO-FRAME_Y | ST_X | ST_Y |
| --- | --- | --- | --- | --- | --- | --- |
| -30 | 44.27 | 44.10 | 45.30 | 43.40 | 42.80 | 46.67 |
| -24 | 45.62 | 43.71 | 45.20 | 44.20 | 44.76 | 44.19 |
| -18 | 43.90 | 42.53 | 43.58 | 41.58 | 45.33 | 42.57 |
| -12 | 46.86 | 42.57 | 46.40 | 43.10 | 44.95 | 42.95 |
| -6 | 46.29 | 42.48 | 47.81 | 40.95 | 46.19 | 46.10 |
| 6 | 47.52 | 45.52 | 46.91 | 53.74 | 46.48 | 46.20 |
| 12 | 44.95 | 46.48 | 52.09 | 51.64 | 44.38 | 44.67 |
| 18 | 44.76 | 46.29 | 49.86 | 55.04 | 42.95 | 39.37 |
| 24 | 45.05 | 47.43 | 50.00 | 52.64 | 43.71 | 45.24 |
| 30 | 42.48 | 50.10 | 52.09 | 50.64 | 42.10 | 46.00 |

Average Jitter results as % wider response

SPSS Output for main analysis

| **Descriptive Statistics** | | | | | | | | |
| --- | --- | --- | --- | --- | --- | --- | --- | --- |
|  | N | Range | Minimum | Maximum | Mean | | Std. Deviation | Variance |
|  | Statistic | Statistic | Statistic | Statistic | Statistic | Std. Error | Statistic | Statistic |
| Luminance | 25 | .0887 | .0305 | .1192 | .050132 | .0036723 | .0183613 | .000 |
| TWO-FRAME | 25 | .0901 | .0401 | .1302 | .061820 | .0042638 | .0213189 | .000 |
| Stereo | 25 | .0692 | .0167 | .0859 | .042304 | .0037217 | .0186087 | .000 |
| Valid N (listwise) | 25 |  |  |  |  |  |  |  |

| **Mauchly's Test of Sphericity^a^** | | | | | | | |
| --- | --- | --- | --- | --- | --- | --- | --- |
| Measure: MEASURE_1 | | | | | | | |
| Within Subjects Effect | Mauchly's W | Approx. Chi-Square | df | Sig. | Epsilon^b^ | | |
|  |  |  |  |  | Greenhouse-Geisser | Huynh-Feldt | Lower-bound |
| factor1 | .932 | 1.612 | 2 | .447 | .937 | 1.000 | .500 |
| Tests the null hypothesis that the error covariance matrix of the orthonormalized transformed dependent variables is proportional to an identity matrix. a. Design: Intercept Within Subjects Design: factor1 b. May be used to adjust the degrees of freedom for the averaged tests of significance. Corrected tests are displayed in the Tests of Within-Subjects Effects table. | | | | | | | |
|  | | | | | | | |
|  | | | | | | | |

| **Tests of Within-Subjects Effects** | | | | | | | |
| --- | --- | --- | --- | --- | --- | --- | --- |
| Measure: MEASURE_1 | | | | | | | |
| Source | | Type III Sum of Squares | df | Mean Square | F | Sig. | Partial Eta Squared |
| factor1 | Sphericity Assumed | .005 | 2 | .002 | 11.779 | <.001 | .329 |
| Error(factor1) | Sphericity Assumed | .010 | 48 | .000 |  |  |  |

A within subjects ANOVA revealed that there was a significant effect of contrast type on discrimination threshold F(2,48) = 11.779, p<.001, PE^2^ = .329

| **^Estimates^** | | | | |
| --- | --- | --- | --- | --- |
| ^Measure: MEASURE_1^ | | | | |
| ^factor1^ | ^Mean^ | ^Std. Error^ | ^95% Confidence Interval^ | |
|  |  |  | ^Lower Bound^ | ^Upper Bound^ |
| ^1^ | ^.050^ | ^.004^ | ^.043^ | ^.058^ |
| ^2^ | ^.062^ | ^.004^ | ^.053^ | ^.071^ |
| ^3^ | ^.042^ | ^.004^ | ^.035^ | ^.050^ |

| **^Pairwise Comparisons^** | | | | | | |
| --- | --- | --- | --- | --- | --- | --- |
| ^Measure: MEASURE_1^ | | | | | | |
| ^(I) factor1^ | ^(J) factor1^ | ^Mean Difference (I-J)^ | ^Std. Error^ | ^Sig.b^ | ^95% Confidence Interval for Differenceb^ | |
|  |  |  |  |  | ^Lower Bound^ | ^Upper Bound^ |
| ^1^ | ^2^ | ^-.012*^ | ^.004^ | ^.011^ | ^-.021^ | ^-.002^ |
|  | ^3^ | ^.008^ | ^.004^ | ^.173^ | ^-.002^ | ^.018^ |
| ^2^ | ^1^ | ^.012*^ | ^.004^ | ^.011^ | ^.002^ | ^.021^ |
|  | ^3^ | ^.020*^ | ^.005^ | ^<.001^ | ^.008^ | ^.031^ |
| ^3^ | ^1^ | ^-.008^ | ^.004^ | ^.173^ | ^-.018^ | ^.002^ |
|  | ^2^ | ^-.020*^ | ^.005^ | ^<.001^ | ^-.031^ | ^-.008^ |
| ^Based on estimated marginal means^ | | | | | | |
| ^*. The mean difference is significant at the .05 level.^ | | | | | | |
| ^b. Adjustment for multiple comparisons: Bonferroni.^ | | | | | | |

Mean difference – Std Error = t value

1 -2 : t=-.012/.004 = -3

1-3: T = .008/.004 = 2

2- 3^:^ t = 0.020/.005 = 4
